# Alleviation of Drought Stress and Metabolic Changes in Timothy (*Phleum pratense* L.) Colonized with *Bacillus subtilis* B26

**DOI:** 10.3389/fpls.2016.00584

**Published:** 2016-05-03

**Authors:** François Gagné-Bourque, Annick Bertrand, Annie Claessens, Konstantinos A. Aliferis, Suha Jabaji

**Affiliations:** ^1^Department of Plant Science, Faculty of Agricultural and Environmental Sciences, Macdonald Campus of McGill University, Sainte-Anne-de-BellevueQC, Canada; ^2^Quebec Research and Development Center, Agriculture and Agri-Food Canada, Québec CityQC, Canada

**Keywords:** amino acids, *Bacillus*, endophytes, osmolytes, timothy grass, plant growth promoting bacteria, sugars

## Abstract

Drought is a major limiting factor of crop productivity worldwide and its incidence is predicted to increase under climate change. Drought adaptation of cool-season grasses is thus a major challenge to secure the agricultural productivity under current and future climate conditions. Endophytes are non-pathogenic plant-associated bacteria that can play an important role in conferring resistance and improving plant tolerance to drought. In this study, the effect of inoculation of the bacterial endophyte *Bacillus subtilis* strain B26 on growth, water status, photosynthetic activity and metabolism of timothy (*Phleum pratense* L.) subjected to drought stress was investigated under controlled conditions. Under both drought-stress and non-stressed conditions, strain B26 successfully colonized the internal tissues of timothy and had a positive impact on plant growth. Exposure of inoculated plant to a 8-week drought-stress led to significant increase in shoot and root biomass by 26.6 and 63.8%, and in photosynthesis and stomatal conductance by 55.2 and 214.9% respectively, compared to non-inoculated plants grown under similar conditions. There was a significant effect of the endophyte on plant metabolism; higher levels of several sugars, notably sucrose and fructans and an increase of key amino acids such as, asparagine, glutamic acid and glutamine were recorded in shoots and roots of colonized plants compared to non-colonized ones. The accumulation of the non-protein amino acid GABA in shoots of stressed plants and in roots of stressed and unstressed plants was increased in the presence of the endophyte. Taken together, our results indicate that *B. subtilis* B26 improves timothy growth under drought stress through the modification of osmolyte accumulation in roots and shoots. These results will contribute to the development of a microbial agent to improve the yield of grass species including forage crops and cereals exposed to environmental stresses.

## Introduction

Timothy (*Phleum pratense* L.) is a perennial forage grass of the Poaceae family widely grown in cool and humid parts of North America and Europe. It is the most important forage grass species in eastern Canada, mainly used for dry hay or silage production. It has numerous important agronomic qualities such as good palatability, high yield in cool and moist conditions, and high winter hardiness making it ideal for regions prone to cold winters (Bélanger et al., 2006). Its shallow and fibrous root system, however, makes it unsuited to dry and/or hot conditions (Marvin and Jerry, 2008). Therefore, the summer regrowth of timothy is often poor and up to 60% of its annual yield is recorded in the first cutting (Lemežienė et al., 2004). With climate change caused by the increase of atmospheric greenhouse gas concentration, Canadian climatic conditions are projected to be warmer with increased accumulated thermal time for plant growth accompanied by a change in the distribution of annual precipitation (Qian et al., 2010). These changes will likely have a major negative impact on timothy regrowth potential (Jing et al., 2014) and thus, on the supply of quality timothy forage to meet the cattle industry dietary needs (Piva et al., 2013). Grazing animals depend on a variety of chemical cues when selecting which plants they will eat, with soluble energy (i.e., free amino acids and organic acids and water-soluble carbohydrates) being the primary factor for selection (Maryland et al., 2000; Tamoura et al., 2009).

Plants face various abiotic stresses among which drought is a major limiting factor of crop growth and productivity. Plants cope with drought stress by different adaptation strategies including physiological, biochemical, and molecular mechanisms (Araus et al., 2002; Chaves et al., 2003; Krasensky and Jonak, 2012). Biochemical mechanisms include the accumulation of compatible osmolytes and antioxidant molecules to help maintain cell turgor pressure, protect cellular macromolecules, membranes and enzyme from oxidative damage (Gill and Tuteja, 2010; Krasensky and Jonak, 2012). A correlation between drought tolerance and accumulation of compatible solutes such as carbohydrates, amino acids and ions to contribute to osmotic adjustments has been documented in grasses (Hanson and Smeekens, 2009; Chen and Jiang, 2010).

Some of the plant growth-promoting bacteria (PGB) have the ability to colonize the internal tissues of plant organs (Hardoim et al., 2008). Because they escape competition with rhizospheric microorganisms and achieve intimate contact with plant tissues, endophytic bacteria are in a unique position among the rhizospheric community to have major interaction with host plants. Irrespective of the mode of colonization, PGB positively influence plant growth or reduce disease and abiotic stresses susceptibility through physical and chemical changes (Dimkpa et al., 2009; Calvo et al., 2014). PGB are adapted to adverse conditions and may help their host-plant to cope with environmental stresses such as drought, and improve plant growth under stress (Vivas et al., 2003; Marulanda et al., 2009, 2010). Various PGB species have been shown to increase drought resistance in wheat, maize, lettuce, and beans (Creus et al., 2004; Figueiredo et al., 2008; Marulanda et al., 2009; Vardharajula et al., 2011; El-Afry et al., 2012; Naveed et al., 2014).

A variety of mechanisms has been proposed to explain stress-induced resistance by PGB in plants (Yang et al., 2009). Some PGB are known to promote root development, thus improving the plant water absorption, likely through the production of phytohormones such as indole acetic acid (IAA), Gibberellic acid (GA), and cytokinins (Boiero et al., 2007; Gagné-Bourque et al., 2015). Others produce 1-aminocyclopropane-1-carboxylate deaminase (Azevedo et al., 2000) that confers induced drought resistance by reducing the production of ethylene (Saleem et al., 2007; Zahir et al., 2008). Some PGB induce modification in plant genes expression, increasing drought-resistance-associated genes like ERD15 (Early Response to Dehydration 15) or DREB (Dehydration Responsive Element Protein) (Timmusk and Wagner, 1999; Gagné-Bourque et al., 2015). In maize, PGB induce metabolic adjustments leading to the accumulation of several organic solutes like soluble sugars, starch and amino acids (Vardharajula et al., 2011).

*Bacillus* is among the most common taxa of isolated endophytes and several strains increase yield and reduce pathogen infection (Lugtenberg and Kamilova, 2009). The proposed mechanisms for plant growth promotion include increased nutrient availability, plant hormones synthesis, and production of volatiles (Ryu et al., 2003; Farag et al., 2006). Information on *Bacillus* strains imparting drought tolerance in plants is limited (Arkhipova et al., 2007; Ashraf and Foolad, 2007; Vardharajula et al., 2011; Wolter and Schroeder, 2012). This is largely because most of the studies concentrated on understanding the mechanisms that elicit plant growth-promoting effects (Dimkpa et al., 2009).

In a previous study, we showed that strain B26 of *B. subtilis* isolated from switchgrass confers drought resistance in *Brachypodium distachyon* through the upregulation of expression of drought-responsive genes, modulation of the DNA methylation process, and an increase in the soluble sugars and starch content of leaves (Gagné-Bourque et al., 2015). Strain B26 was also shown to form intimate association with host plants and to produce several well-characterized lipopeptide toxins and phytohormones (Gagne-Bourque et al., 2013). These characteristics suggest that strain B26 has a strong potential as bio-inoculant for enhancement of biomass of grasses and plants’ defense against abiotic stresses such as drought.

The aim of this work was to explore the potential for improving growth and physiological responses of timothy under an extended drought stress period in association with *B. subtilis* strain B26. Thus, we focused our efforts on studying the fluctuation of soluble sugars, starch and amino acids since these primary metabolites are important for quality of cool-season forage crops in terms of palatability and digestibility for grazing animals. To our knowledge, this is the first comprehensive analysis of shoot and root metabolite changes in response to drought stress in timothy associated or not with an endophyte.

## Materials and Methods

### Maintenance and Preparation of *Bacillus subtilis* B26 Inoculum

The fully characterized *Bacillus subtilis* strain B26, was maintained on standard microbiological medium following the method of Gagne-Bourque et al. (2013). Inoculum consisted of 10^6^ colony forming units (CFU) mL^-1^ in phosphate buffer (Yasbin et al., 1975) and was prepared as previously described (Gagne-Bourque et al., 2013).

### Plant Material and Growth Conditions

**FIGURE 1:**
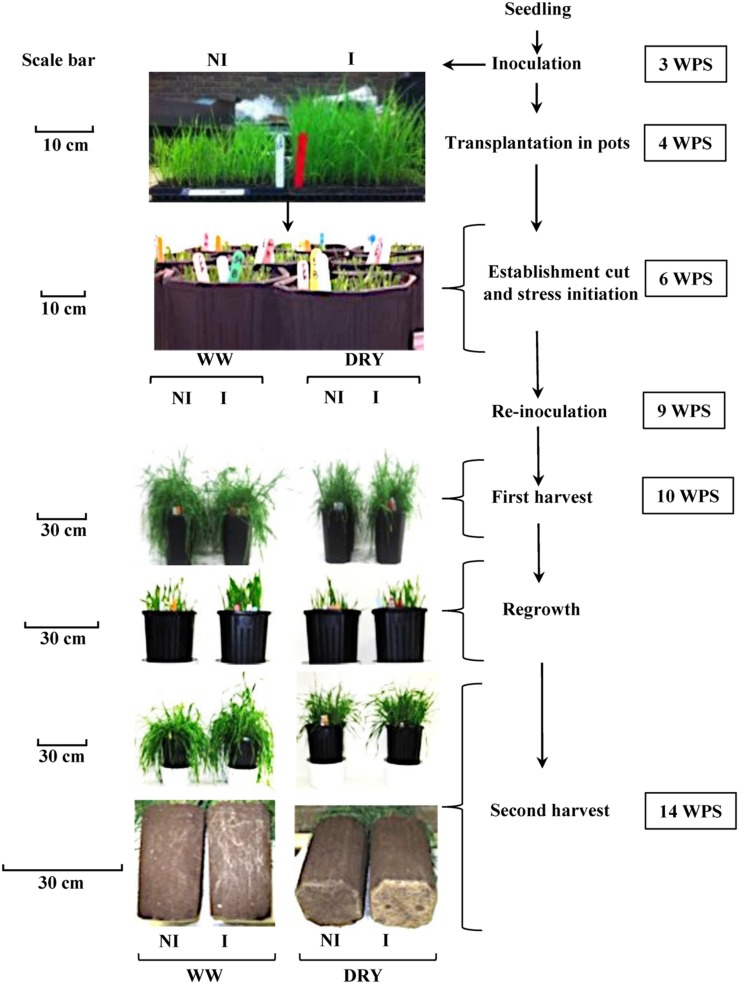
**Flow chart of the experimental set-up of timothy inoculated (I) or non-inoculated (NI) with *Bacillus subtilis* B26, grown under well- watered (WW) or water-stressed conditions (DRY).** The right panel describes the timing of each step expressed as weeks post seeding (WPS). The left panel presents pictures of the plants at different key steps under each treatment. Scale in each sub-panel represents the plant’s shoot height. Scale in the last sub-panel represents the depth of soil column.

The experiment was conducted under controlled conditions between July 23th and October 29th, 2014 at the Agriculture and Agri-Food Canada Research Centre in Québec City, QC, Canada. Seeds (cv Novio) were planted individually in microcell trays (1.5 cm × 1.5 cm × 3 cm) (The Blackmore Company, Belleville, MI, USA) containing a soil mixture of commercial topsoil:perlite (Holiday perlite; V.I.L Vermiculite Inc., Lachine, QC, Canada):peat moss (Pro-mix BX; Premier Peat Moss, Rivière-du-Loup, QC, Canada) (10:1:1, v/v/v). A total of 2048 microcells were planted. The soil mixture was autoclaved for 3 h at 121°C for three constitutive days prior to planting. Plants were transferred in four growth chambers (Conviron, Model PGR15, Controlled Environments Limited, Winnipeg, MB, Canada) under the following conditions: 16 h photoperiod with a day/night temperature regime of 20/10°C. Seedlings were watered as needed. The flow chart of the experimental set-up described below is illustrated in **Figure 1**.

At 3 weeks post seeding (WPS), each seedling was inoculated by pipetting 0.5 mL of phosphate buffer containing 10^6^ CFU of *B. subtilis* in the soil surrounding each plant in the tray (**Figure 1**). Non-inoculated seedlings (Control) received 0.5 mL of sterilized phosphate buffer. Re-inoculation of plants with strain B26 (1 mL) was performed 9 WPS following the same procedure.

At four WPS individual plants from the microcells were transplanted in 32-cm deep pots (10 plants/pot) (TPOT3, Stuewe and Sons, Tangent, OR, USA) containing 4 kg of the soil mixture previously described. Pots containing inoculated and non-inoculated plants were transferred in four separate growth chambers (two for inoculated plants and two for non-inoculated plants). Pots were rotated every week between the two growth chambers dedicated to the same inoculation treatment in order to avoid confounding treatment effects with chamber effects (Bertrand et al., 2014).

Following a 2-week establishment period (i.e., 6 WPS), plants were cut at 3-cm height (establishment cut) and the following four treatments were applied: (i) inoculated and well-watered (WW) (ii) non-inoculated and WW; (iii) inoculated and water-stressed and (iv) non-inoculated and water-stressed. WW plants were watered to field capacity three times per week based on pot weight. Pots of water-stressed plants (DRY) received ¼ of the water amount added to WW plants. All pots received 100 mL of a solution of 1 g L^-1^of N-P-K fertilizer 20-20-20 (Plant Products, Laval, QC, Canada) once a week. Air temperature in the growth chamber was set to a day/night temperature regime of 25/15°C under a 16-h photoperiod.

A first destructive harvest (H1) was performed on half of the plants after 4 weeks of water treatment (i.e., 10 WPS) when approximately 80% of the plants reached the early anthesis stage (Simon and Park, 1983). The remaining pots were cut at 3 cm-height and then left to regrow for an additional 4 weeks after which a second harvest (H2; 8 weeks of water treatment) was performed. These sequential harvests were used in order to simulate the standard cut management practice for timothy in the field (**Figure 1**). At each harvest, destructive measurements were taken from eight pots for each of the four-treatment combination described above. Half of these pots were used for the measurement of photosynthesis and stomatal conductance, biomass yield of roots and shoots, and biochemical analyses. The remaining four pots were used for the measurement of soil moisture, plant water content, and microbiological and molecular tests. A total of 64 pots were used at each harvest and pots were placed in a complete randomized block design.

### Physiological and Biochemical Analyses

#### Plant Biomass

At each harvest (H1 and H2), the above ground biomass of plants in each pot was collected and the remaining roots and stubble were thoroughly washed to remove all traces of soil. Forage and root biomass were dried at 55°C for 72 h, weighed and ground to pass a 1-mm screen with a Wiley mill (model 3379-k35, Variable Speed Digital ED-5 Wiley Mill, Thomas Scientific, Swedesboro, NJ, USA). Ground samples were stored in 90 mL screw cap containers (Thermo Fisher Scientific, Ottawa, ON, Canada) at 22°C for analyses of carbohydrates and amino acids. Four biological replicates, each composed of 10-pooled plants, were used for photosynthesis and conductivity measurement.

The photosynthetic rate and stomatal conductance were measured on the youngest fully developed leaf of a representative tiller from each pot using the LI-6400XT portable photosynthesis system (LI-COR, Lincoln, NE, USA). Measurements were made at room temperature between 10:00 and 14:00 h under a PPFD of 1000 μmol m^-2^ s^-1^ provided by a red and blue light source (Model 6400-02B, Li-COR) and vapor pressure deficits varying from 1.5 to 2.5 kPa.

#### Leaf Water Potential

Two representative non-flowering tillers per pot were selected and cut below the fourth youngest mature leaf to determine the leaf water potential using a portable pressure chambers (3005F01 Plant Water Status Console, Soil Moisture Equipment Corp., Santa Barbara, CA, USA).

### Assessment of Plant Colonization by *Bacillus subtilis* B26

The abundance and systemic internal colonization of timothy tissues by B26 was confirmed by culture-dependent (CFU counts) and culture-independent methods (DNA copy numbers), and also in rhizospheric soil of inoculated and non-inoculated plants subjected or not to water stress. At each harvest, four plants/pot of each replicate of all treatments were randomly selected and shoots and roots were separated and pooled. Roots were gently shaken to collect rhizosphere soil. Irrespective of the method applied, all collected tissue samples were surface sterilized following a stepwise protocol of ethanol, sodium hypochlorite and water (Gagne-Bourque et al., 2013).

Homogenized tissue samples (200 mg) and rhizospheric soil (1 g) from WW and DRY treatments, inoculated or not, were serially diluted in phosphate buffer, plated on LBA (Skinner et al., 1952), and incubated at 37°C for 24 h. CFU were determined and calculated to Log CFU per gram of fresh weight of tissue or soil. There were four biological replicates each consisted of four plants for each treatment. Root tissues of H2 were lignified and impossible to properly homogenize, and thus were not subjected to bacterial enumeration. The presence of *B. subtilis* B26 cells inside inoculated plants subjected or not to water stress was also confirmed by quantitative real-time PCR (qPCR) assays. Plant Genomic DNA was extracted using the CTAB method (Porebski et al., 1997) and genomic DNA from *B. subtilis* B26 colonies was extracted using the direct colony PCR (Woodman, 2008). *B. subtilis* B26 amplicons from strain specific primers (Gagne-Bourque et al., 2013) were purified, cloned and used to build a standard curve for qPCR assays (Gagné-Bourque et al., 2015).

### Extraction of Carbohydrates and Amino Acids and Starch Quantification

At each harvest, approximately 200 mg of dried shoots and roots was incubated in 7 mL of deionised H_2_O at 80°C for 20 min to stop enzyme activity. To optimize sugar and amino acids extraction, tubes were incubated overnight at 4°C. After a 10 min centrifugation at 1500 × *g*, 1-mL sub-sample of the supernatant was collected for quantification of soluble carbohydrates and free amino acids. The pellets were used for starch quantification following an enzymatic digestion with amyloglucosidase (Sigma A7255; Sigma-Aldrich Co., St. Louis, MO, USA). Starch was quantified as glucose equivalents by a colorimetric detection with hydrobenzoic acid hydrazide method of Blakeney and Mutton (1980) on a spectrophotometer set to a wavelength of 415 nm.

#### Soluble Sugars and Low Degree of Polymerization Fructans

Soluble sugars including sucrose, glucose, fructose, raffinose and low degree of polymerization (LDP) fructans were diluted 1:1 ratio with acetonitrile and centrifuged for 3 min at 16,000 × *g* and kept at 4°C in the sample manager throughout the analysis. Waters ACQUITY Ultra Performance Liquid Chromatography (UPLC) analyzer controlled by the Empower II software (Waters, Milford, MA, USA) was used for the chromatographic analyses. Samples were separated on a BEH Amide Acquity UPLC column (Waters, Milford, MA, USA) and detected on an Electric Light Scattering Detector (ELSD, Acquity, Waters) set to a gas pressure of 45 psi. The chromatic conditions were as follows: 0.25 mL min^-1^ with a gradient of eluents A (80% acetonitrile/0.1% NH_4_OH) and B (30% acetonitrile/0.1% NH_4_OH) described in Piva et al. (2013). The drift tube was adjusted to a temperature of 50°C in the cooling mode. Peak identity and quantity of soluble sugars were determined by comparison to analytical standards following the guidelines of the metabolomics standards initiative (Fiehn et al., 2007). The degree of polymerization of LDP fructans was established by comparison with retention times (**Supplementary Table S1**) of purified standards from Jerusalem artichoke (*Helianthus tuberosus* L.).

#### High Degree of Polymerization Fructans

High degree of polymerisation fructans (HDP), from DP 10 to DP 200 were separated on a Shodex KS-804 column preceded by a Shodex KS-G pre-column (Shodex, Tokyo, Japan) and detected on a Waters 2410 refractive index detector following these chromatographic conditions: isocratic elution at 50°C with deionized water at a flow rate of 1.0 mL min^-1^ (Hofmann et al., 2014). The degree of polymerization of fructans was determined by reference to polymaltotriose pullulan standards (Shodex Standard P-82) and concentrations of both LDP and HDP fructans was expressed as fructose equivalent.

#### Amino Acids

Amino acids were derivatized using the AccQ Tag Ultra reagent (6-aminoquinolyl-*N*-hydroxysuccinimidyl carbamate) and separated on an AccQ Tag Ultra column (2.1 mm × 100 mm). The 21 amino acids were detected with Waters ACQUITY Tunable UV detector (Waters) at 260 nm under previously described chromatographic conditions (Cohen, 2000). The Waters ACQUITY UPLC analytical system was controlled by the Empower II software. Peak identity and quantity were determined by comparison to analytical standards (**Supplementary Table S1**), and results were expressed as concentrations on dry weight basis (μmol g^-1^ DW).

### Statistical Analyses and Biomarker Discovery

One-way ANOVA was performed using the JMP 10.0 software (SAS Institute, Cary, NC, USA) on phenotypic measurements (i.e., biomass, photosynthesis rate, stomatal conductance, water potential, and soil moisture), and on microbial abundance (CFU numbers and DNA copies). All experimental data were tested for statistical significance using Tukey HSD with a magnitude of the *F*-value (*P* = 0.05). Although each harvest was analyzed separately with no interaction effect between different treatments and harvest time (*F* = 0.05), the data were presented as two separate analyses.

Multivariate analysis was performed on the carbohydrate and amino acids contents for the detection of trends and metabolite-biomarkers as previously described (Aliferis et al., 2014). Briefly, datasets were subjected to multivariate analyses using the SIMCA-P+ v.12.0 software (Umetrics, MKS Instruments Inc. Umeå, Sweden) for the dissection of the effects of B26 inoculation and drought on the metabolism of the plant. For the overview of the datasets and the detection of possible outliers, principal component analysis (PCA) was initially performed. Additionally, a heatmap was constructed using the programming language R for the robust visualization of metabolite level fluctuations among treatments. The detection of biomarkers was based on orthogonal projections to latent structures-discriminant analysis (OPLS-DA) regression coefficients (*P* < 0.05). Corresponding standard errors were calculated using Jack-knifing with 95% confidence interval. The performance of the obtained models was assessed by the cumulative fraction of the total variation of the *X*’s that could be predicted by the extracted components [Q_(cum)_^2^; cumulative fraction of the total variation of the *X*’s that can be predicted by the extracted components, *R^2^X* and *R^2^Y*, the fraction of the sum of squares of all *X*’s and *Y*’s explained by the current component, respectively].

## Results and Discussion

### Stable Colonization of Timothy by B26

**FIGURE 2:**
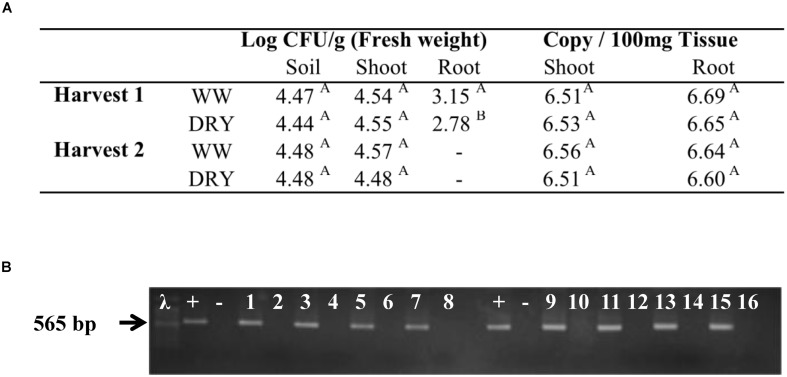
**Assessment of plant and soil colonization by *B. subtilis* B26 after 4 weeks (harvest 1) and 8 weeks (harvest 2) of WW or water stressed (DRY) treatments presented by **(A)** the estimation of colony forming units (CFU) in rhizosheric soil, and in timothy shoots and roots and DNA copy number of *B. subtilis* B26 in timothy shoot and root tissues. (B)** Electrophoresis gel showing qPCR amplification of putative products amplified by species-specific primers: Lane +, *B. subtilis* B26 pure DNA; Lane -, no template; Lanes 1, 3, 5, and 7, DNA templates from inoculated shoots; Lanes 2, 4, 6, and 8, DNA templates from non-inoculated shoots; Lanes 9, 11, 13, and 15, DNA templates from inoculated roots; Lanes 10, 12, 14, and 16, DNA templates from non-inoculated roots.

A stable colonization of timothy by *B. subtilis* strain B26 was necessary to ensure the success of the experiment. The process for bacterial endophytes to colonize plants is complex; it requires resistance to plant defense mechanisms as well as the ability to initiate growth on plant surfaces, and to develop inside the plants (Truyens et al., 2014). In this study, the strain B26 efficiently colonized the rhizosphere and timothy roots and was also intimately associated with the plant since it was isolated from the interior of root and shoot tissues of inoculated plants at both harvests. The success of internal and systemic colonization of timothy by B26 was confirmed by culture-dependent (**Figure 2A**) and independent methods (**Figure 2B**). Re-isolation and quantification of strain B26 by the plating method in both tissues of WW and DRY plants clearly demonstrate that it can form sustainable, endophytic populations in roots, shoots as well as in the soil around the roots of timothy (**Figure 2A**). The presence of *B. subtilis* B26 in different tissues of timothy was confirmed by qPCR in inoculated plants (**Figure 2B**). Furthermore, an amplicon with the expected product size of 565 bp was successfully amplified using species-specific primers for *B. subtilis* B26 from DNA extracted from each tissue type (**Figure 2B**). Population numbers of B26 in soil and in timothy shoot and root tissues were similar, ranging from log_10_ 4.44 to 4.57 log_10_ CFU at both harvests and so were the absolute DNA copy numbers, which were sustained in roots and shoots. These densities are comparable to what has been reported for *Bacillus* species including *B. subtilis* in soil and in other plant species (van Elsas et al., 1986; Rai et al., 2007; Ji et al., 2008; Liu et al., 2009). Taken together, these results indicate that timothy colonization potential of the strain was not hampered with water stress conditions.

### Physiological Response of B26-Colonized Timothy to Drought

**FIGURE 3:**
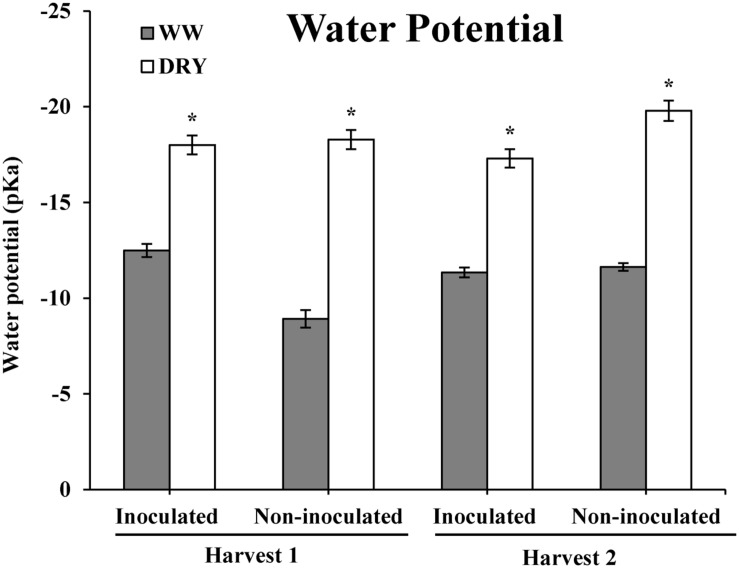
**Water potential (bars ± SE) of timothy plants inoculated and non-inoculated with *B. subtilis* B26 after 4 weeks (harvest 1, H1) and 8 weeks (harvest 2, H2) under WW or DRY conditions.** * Indicates significance at the 0.05 probability level.

The goal of this study was to characterize the drought-stress response of timothy in association with the endophyte *B. subtilis*, strain B26 and to determine if the bacteria could improve plant drought tolerance. Results show that reducing plant watering by 75% throughout the experiment significantly reduced the water potential of timothy from -12 bar in WW plants to -18 bars in DRY plants at both harvests showing the successful establishment of a water stress in DRY as compared to WW treatments (**Figure 3**).

**FIGURE 4:**
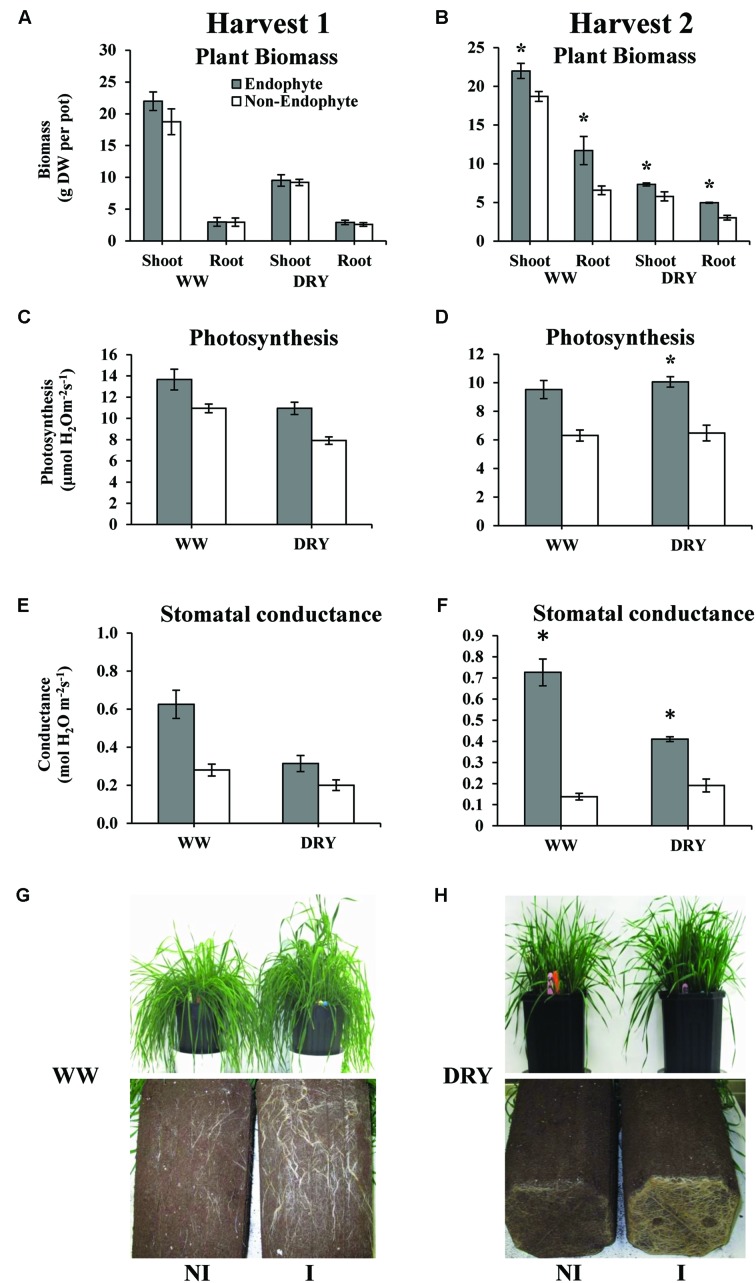
**Biomass in grams dry weight (DW) per pot, of shoot and root **(A,B)**, photosynthetic rate **(C,D)** and stomatal conductance **(E,F)** of timothy inoculated or non-inoculated with *B. subtilis* B26 after 4 weeks (harvest 1; **A,C,E**) and 8 weeks (harvest 2; **B,D,F**) under WW or DRY conditions.** Error bars represent the standard error of the mean. **(G)** Roots and shoots of non-inoculated (NI) and inoculated (I) plants at harvest 2 of WW treatment; **(H)** Roots and shoots of non-inoculated (NI) and inoculated (I) plants at harvest 2 of DRY treatment. *Indicates significance at the 0.05 probability level for *n* = 4.

Our results clearly show that inoculation with endophytic *B. subtilis* strain B26 significantly increased root and shoot biomass under both WW and DRY conditions at second harvest (**Figure 4B**). Under DRY conditions, shoot dry biomass was increased by 27% and root biomass by 64% in the presence of endophytes (**Figure 4B**). Experiments conducted in pots do not always allow for the assessment of water-treatment effect on root growth due to pot-bound limitation. In order to avoid this issue, we used deep pots (32 cm-deep) allowing for optimal root growth throughout the experiment. This set-up allowed us to observe a major positive effect of the endophytes on root growth under drought stress. *Bacillus* spp. has been shown to colonize the rhizosphere and was reported to promote growth and enhance biotic and abiotic stress tolerance in a number of crops by different mechanisms including the mobilization of soil nutrients and the synthesis of phytohormones (Saleem et al., 2007; Hardoim et al., 2008). As we have previously reported, growth stimulation of timothy by *B. subtilis* strain B26 is likely due to the solubilisation of P and the production of indole-3- acetic acid (IAA) and the cytokinin zeatin riboside by this strain (Gagne-Bourque et al., 2013).

One of the first responses of plants to drought stress is the reduction of stomatal conductance in order to decrease water loss by transpiration (Medrano et al., 2002). Stomatal closure has the disadvantage of limiting the entry of CO_2_ and reducing the photosynthetic rate. To maintain growth under drought, a compromise between carbon assimilation and water transpiration has to be reached. In the present study, we observed that inoculation with *B. subtilis* B26 resulted in higher photosynthetic rate by 55% under DRY conditions at second harvest (**Figure 4D**). Accordingly, stomatal conductance was significantly higher (nearly four times higher under WW and 2.5 times higher under DRY) in the presence of endophytes (**Figure 4F**). Under DRY conditions, *B. subtilis*-inoculated timothy had a greater root biomass, which might partially explain the observed higher conductance compared to the non-inoculated plants. Increase in total root biomass under stress conditions is the most commonly reported plant response mediated by PGB inoculation in various crops (Wani and Khan, 2010; Kasim et al., 2013). Investing more energy in developing a larger root system in order to optimize water extraction and minimizing water loss is a well-known drought avoidance mechanism by which plants manage to delay the consequence of drought (Chaves et al., 2003; Meister et al., 2014). These observations are in agreement with previous reports on the potential of endophytic bacteria in improving plant productivity and enhancement of drought tolerance (Creus et al., 2004; Mei and Flinn, 2010).

### Metabolic Response of Inoculated Plants to Drought Stress

#### Analysis Overview

As previously mentioned, plants cope with drought stress by biochemical mechanisms including the accumulation of compatible solutes and other key stress-induced metabolites (Chen and Jiang, 2010; Krasensky and Jonak, 2012). To characterize the metabolic responses of timothy to drought stress and the potential effect of the endophyte *B. subtilis* B26 on this response, we assessed the differences in metabolite accumulation in shoots and roots of inoculated or non-inoculated timothy plants under WW and DRY conditions (**Supplementary Table S2**). Analyses revealed a strong discrimination between inoculated and non-inoculated plants, between the watering levels (DRY and WW) and between the two harvests (H1 and H2) (**Supplementary Figure S1**). The study of trends in the data sets and the discovery of metabolites-biomarkers performing pairwise comparisons of the treatments were based on multivariate analyses. Representative chromatograms are displayed in the **Supplementary Figures S2** and **S3**.

**FIGURE 5:**
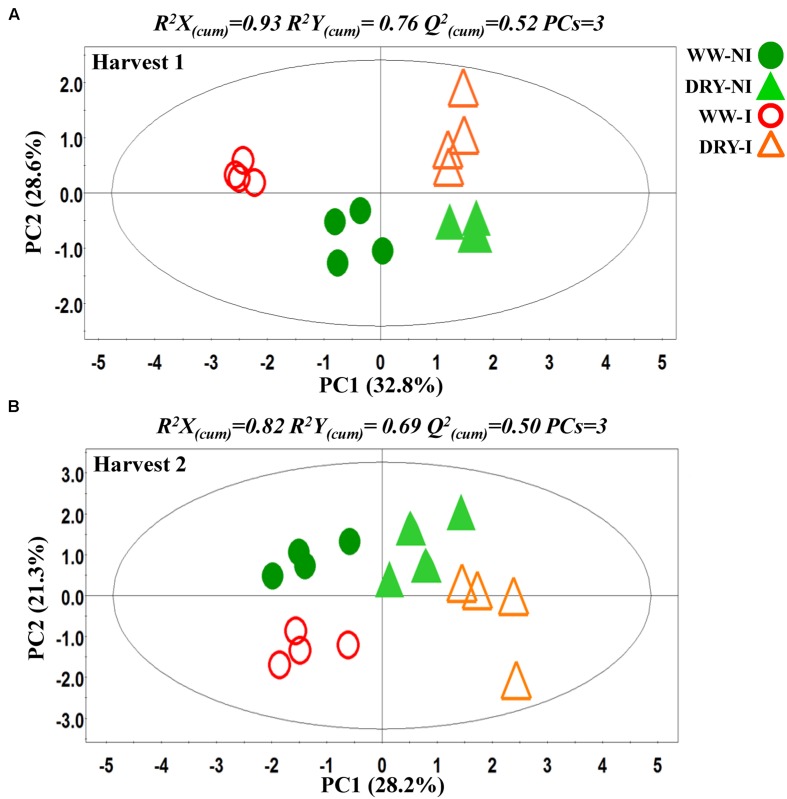
**Partial least squares-discriminant analyses (PLS-DA) PC1/PC2 score plots for **(A)** harvests 1 (H1) and **(B)** harvest 2 (H2).** The ellipse represents the Hotelling T^2^ with 95% confidence interval. Four biological replications each consisting of ten plants were performed per treatment [Q_(cum)_^2^; cumulative fraction of the total variation of the *X*’s that can be predicted by the extracted components, *R^2^X* and *R^2^Y*; the fraction of the sum of squares of all *X*’s and *Y*’s explained by the current component, respectively]. WW; well-watered, DRY; dry, NI; no inoculation with B26 I; inoculation with B26.

**FIGURE 6:**
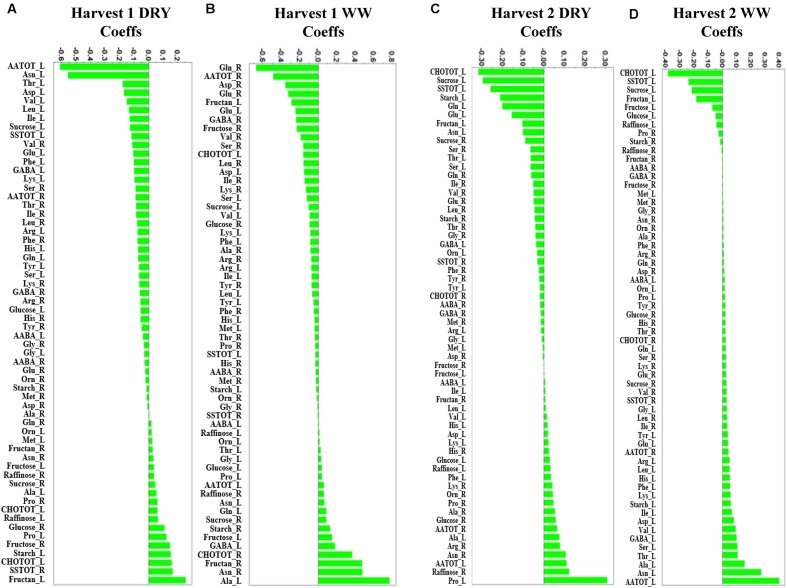
**Orthogonal projections to latent structures-discriminant analysis (OPLS-DA) coefficient (Coeffs) plots for the comparisons between inoculated and non-inoculated with *B. subtilis* B26 timothy plants [shoots (L) and roots (R)] after 4 weeks (Harvest 1) under **(A)** water stress (DRY) and **(B)** WW conditions and after 8 weeks (Harvest 2) under **(C)** water stress (DRY) and **(D)** WW conditions (*P* = 0.05).** Negative Coeffs values represent metabolites with higher concentrations in inoculated plants whereas positive values correspond to those with higher concentration in non-inoculated plants (Ala, alanine; Arg, arginine; Asn, asparagine; Asp, aspartic acid; Gln, glutamine; Glu, glutamic acid; Gly, glycine; His, histidine; Ile, isoleucine; Leu, leucine; Lys, leucine; Lys, lysine; Met, methionine; Phe, phenylalanine; Pro, proline; Ser, serine; Thr, threonine; Tyr, tyrosine; Val, valine; Orn, Ornithine; AATOT, Total amino acid; SSTOT, total soluble sugars; CHOTOT, total carbohydrates; AABA, *α*-aminobutyric acid; GABA, *γ*-aminobutyric acid).

**FIGURE 7:**
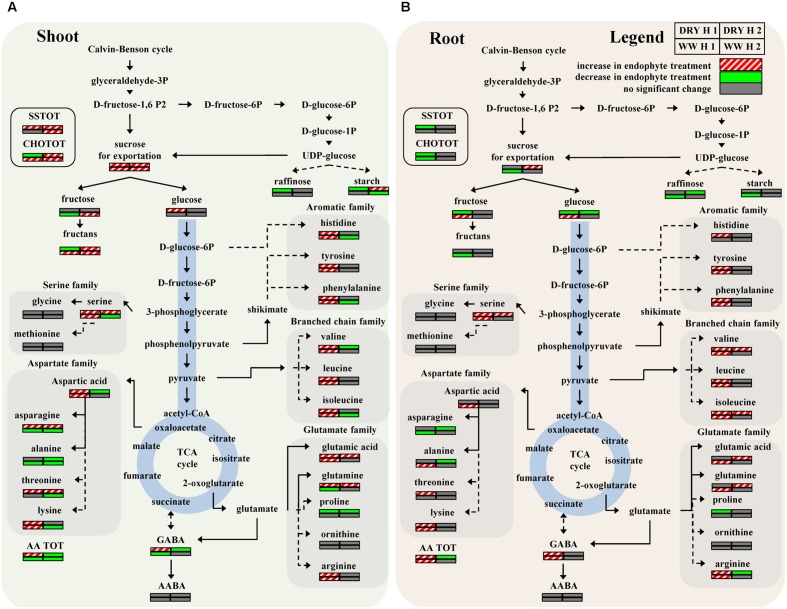
**Metabolic pathway maps of changes in metabolite concentration induced by endophyte colonization in timothy shoots **(A)** and roots **(B)** after 4 weeks (H1) and 8 weeks (H2) under WW or DRY conditions.** Variable relative concentrations (increased, decreased or no change in response to endophytes) are coded using a color based on the means of scaled and centered OPLS regression coefficients from 4 biological replications. Dashed arrows symbolize a multistep and solid arrows a one-step reactions (for abbreviations consult the legend of **Figure 6**).

**FIGURE 8:**
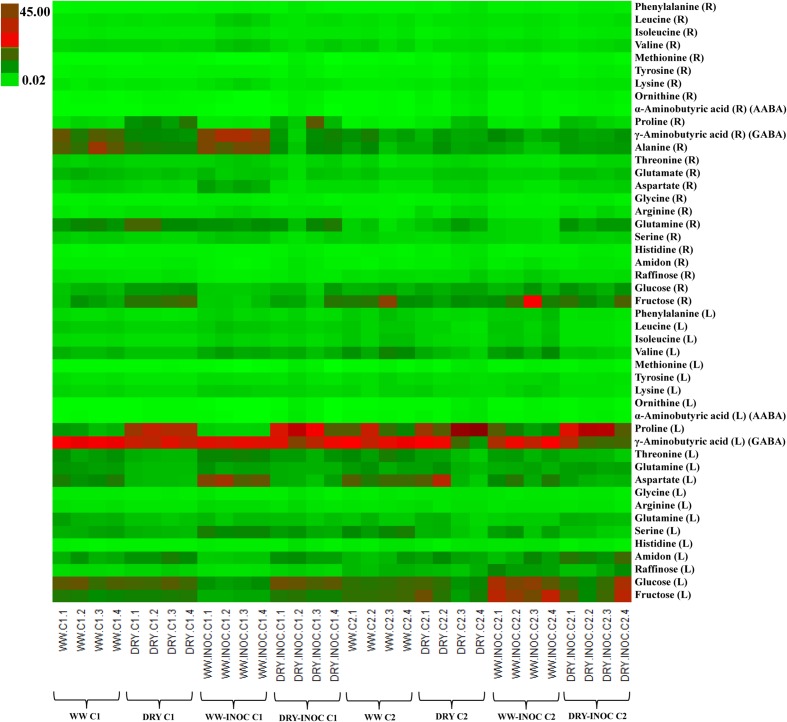
**Heatmap with color-coded concentrations of identified timothy metabolites expressed as mg g^-1^ of dry matter (WW; well-watered, DRY; water-stressed, INOC; *B. subtilis* B26-inoculated plants, L; shoots, R; roots)**.

PCA was initially performed on the two datasets revealing no outliers (*P* < 0.05, data not shown). In a second step, orthogonal projections to latent structures-discriminant analysis (OPLS-DA) with regression coefficients (*P* < 0.05) were employed for the different treatments at H1 and H2 (**Figures 5A,B**). The obtained OPLS-DA score plots revealed a strong effect of the water condition and of B26 inoculation on the metabolic composition of the plants. Furthermore, the tight clustering among biological replications and absence of outliers, confirm the robustness and reproducibility of the experimental bioanalytical and bioinformatics protocols (**Figures 5A,B**). The corresponding metabolites-biomarkers of the plant response to B26 inoculation under DRY and WW conditions for the H1 and H2, are displayed in the corresponding coefficient plots (**Figures 6A–D**) and in the metabolic pathway maps of changes in metabolite concentration (**Figure 7**). Complementary to multivariate analysis, the constructed heatmap revealed the patterns of fluctuation of metabolite levels among treatments, enabling an overview of the effect of the various treatments on the metabolism of the plant (**Figure 8**).

**FIGURE 9:**
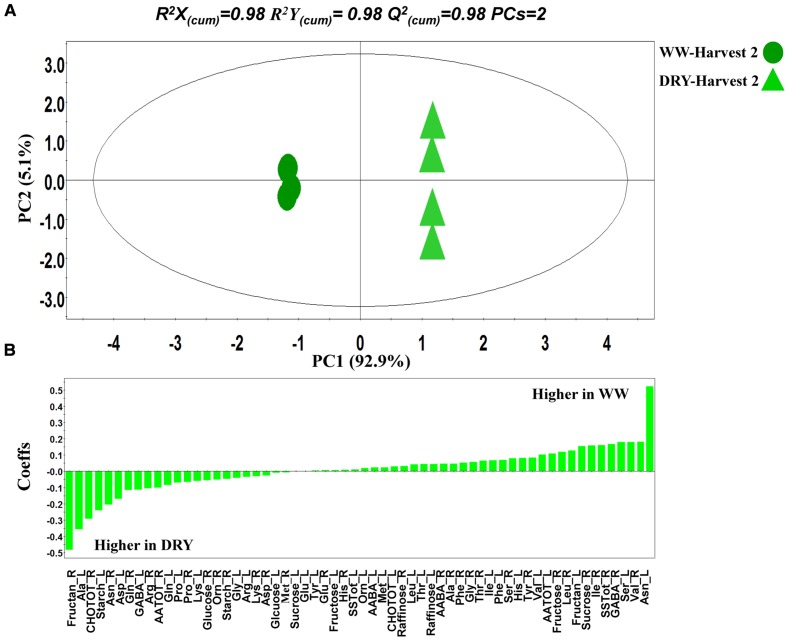
**Partial least squares-discriminant analyses (PLS-DA) PC1/PC2 score plot **(A)** and corresponding coefficient (Coeffs) **(B)** plot for the comparison between timothy plants grown under WW and water-stressed (DRY) conditions.** The ellipse represents the Hotelling T^2^ with 95% confidence interval. Four biological replications each consisting of ten plants were performed per treatment [Q_(cum)_^2^; cumulative fraction of the total variation of the *X*’s that can be predicted by the extracted components, *R^2^X* and *R^2^Y*; the fraction of the sum of squares of all *X*’s and *Y*’s explained by the current component, respectively].

OPLS-DA was also performed for the discovery of biomarkers of plant metabolic response to drought in the absence of B26 inoculation (**Figure 9A**). The corresponding metabolite-biomarkers of drought response are displayed in **Figure 9B**. Results of the above two comparisons are discussed in details in the next sections.

The assignment of the origin of metabolites in plant-microbe interactions (i.e., plant-pathogen or plant-beneficial microbe interactions such as the one described here) is challenging in metabolomics studies. Bacterial endo- and exo-metabolomes contain several primary and secondary metabolites common to those of plants. However, here, the leverage of the bacterial metabolites on the total recorded profile is minimal as it can be supported by the fact that the patterns of fluctuation for common identified plant metabolites are independent of the treatments (e.g., inoculated or non-inoculated plants) as shown in **Figure 7**.

#### Sugars

Soluble sugars such as, sucrose, glucose, fructose, and raffinose are among metabolites that typically accumulate under drought stress in plant leaves (Hare et al., 1998; Bowne et al., 2012). These sugars affect osmotic adjustment, and help to maintain homeostasis allowing the plant to preserve its turgor pressure and thus, its normal functions under water-limiting environment (Krasensky and Jonak, 2012).

Drought stress is known to disrupt carbohydrate metabolism and to decrease sucrose level in leaves, presumably due to an induced increase of invertase activity (Ruan et al., 2010). This may hamper the rate of sucrose export to the sink organs. In our study, bacterized plants generally accumulated more total carbohydrates and total soluble sugars in shoots compared to roots of non-stressed and stressed plants (**Figures 6** and **7**). Inoculation of timothy with strain B26 increased most notably sucrose and fructan concentrations in shoots under non-stressed and drought-stressed conditions over a period of 8 weeks of drought, while glucose increased in plants after 4 weeks of drought stress (**Figures 6** and **7A**). Such increases were directly linked to the presence of strain B26, which shows that *B. subtilis* contributed to increase the biosynthesis of sugars to allow for better osmotic adjustment and thus alleviates stress effect on host plant. Furthermore, since sucrose concentration increased in both shoots and roots, we could conclude that sucrose export was not reduced under drought stress conditions in the presence of B26. Similar results have been reported in other bacterial-endophyte systems. Bacterial endophytes enhanced drought and cold tolerance of tall fescue, maize and grapevine plants with higher and faster accumulation of stress-related metabolites (Vardharajula et al., 2011; Fernandez et al., 2012; Nagabhyru et al., 2013).

When looking exclusively at the effect of drought stress on sugar accumulation, we observed a marked increase of fructans and of total carbohydrates in roots while it decreased in shoots, showing an enhancement of carbohydrate allocation to the roots under drought. The allocation of dry matter and carbohydrates to roots is considered as a drought adaptation that enhances plant water uptake (De Souza and Da Silva, 1987; Leport et al., 1999; Farooq et al., 2009). Our results show that the presence of the endophyte further contributed to drought adaptation in timothy by increasing the biomass of the root system (**Figures 1** and **4**) and by increasing the concentration of the osmotically active sugar sucrose in roots after 8 weeks of drought (**Figure 9B**).

#### Amino Acids

During water stress, protein synthesis is slowed and hydrolysis may occur, promoting an increase in soluble nitrogen compounds such as free amino acids (Farooq et al., 2009; Krasensky and Jonak, 2012). Levels of amino acids have been shown to increase in drought stressed plants (Bowne et al., 2012). A total of 21 amino acids were measured in shoots and roots of WW and drought-stressed timothy plants inoculated or not with B26. Many amino acids from the aromatic, and the glutamate and aspartate families were produced in greater quantities in shoots and roots of plants inoculated with *B. subtilis* B26 under drought-stress conditions, particularly at the first harvest after 4 weeks (**Figures 6** and **7**). For instance, total amino acids increased markedly in shoots under drought conditions in the presence of B26 at harvest 1 while the reverse was observed at harvest 2 with a sharp decrease of total amino acids in shoots. To a lower extent, we observed a similar transient increase of total amino acids in roots under drought and WW conditions showing that this effect is mostly linked to the B26 inoculation. Early report from (Lyons et al., 1990) documented an increase in amino acid concentrations in shoots of tall fescue when inoculated with endophytes and they concluded that, in addition to the synthesis by the plant, endophytes could have directly contributed to this higher level of amino acids. As in our study, they observed that the largest change in amino acid concentration was due to a large increase of asparagine in shoots of bacterized plants. A larger sink for this amino acid in the shoots where it could have contributed to the metabolism of the endophyte could explain the concomitant decrease of this major transport amino acid in the roots at both harvests.

#### Aromatic Amino Acids Family

The increased levels of histidine, tyrosine and phenylalanine were consistent in bacterized timothy plants exposed to a 4 week-period of water deficit or in WW plants (**Figures 6** and **7**). These aromatic amino acids have been implicated in drought-stress response in maize and wheat (Harrigan et al., 2007; Witt et al., 2011; Bowne et al., 2012). Histidine, an essential amino acid required for plant growth and development, functions as a metal-binding ligand and as a major part of metal hyperaccumulator molecule leading to alleviation of heavy metal stress (Sharma and Dietz, 2006), and has also been reported to play a role in abiotic stress resistance (Harrigan et al., 2007). Tyrosine and phenylalanine are synthesized through the shikimate pathway and serve as precursors for a wide range of secondary metabolites, including reactive oxygen species (ROS) scavengers (Less and Galili, 2008; Gill and Tuteja, 2010). Water deficit enhances the production of ROS and the maintenance or increase in the activity of enzymes involved in removing toxic ROS to avoid cellular damage is regarded as an important factor in tolerance to dehydration (Chaves et al., 2003).

#### Branched Chain Amino Acids Family

Valine, leucine and isoleucine, the branched amino acids increased in shoots and roots of bacterized timothy plants (**Figures 6** and **7**), however, their accumulation was mostly prominent in shoots of bacterized plants exposed to 4-week period of stress (**Figures 6** and **7**) and in roots of bacterized plants exposed to an 8-week period of stress (**Figure 6**). These results support that branched amino acids play an active role in plant drought tolerance or avoidance mechanism, as was previously reported in wheat and peas (Charlton et al., 2008; Bowne et al., 2012). Taylor et al. (2004) stated that branched amino acids might provide a source of energy in sugar starved *Arabidopsis*, while Joshi and Jander (2009) proposed that they can act as osmolytes thus increasing *Arabidopsis* drought tolerance.

#### Aspartate Family

Aspartic acid, asparagine, threonine and lysine accumulate in a range of plant tissues under stress (Barnett and Naylor, 1966; Kusaka et al., 2005; Lea et al., 2007). Most notably was the considerable accumulation of asparagine in shoots of bacterized plants exposed to an extended 8 week-period of stress that likely originated from the roots (**Figures 6C,D** and **7**). The further steps of the metabolic pathways of the aspartate family amino acids do not seem to be linked with endophyte inoculation since we observed no consistent endophyte effects on these amino acids levels nor on their translocation between shoots and roots. A similar trend was reported for water stressed tall fescue infected with the fungal endophyte *Neotyphodium coenophialum* (Nagabhyru et al., 2013).

#### Glutamate Family

Proline is a well-known marker of water and salt stresses in plants. It is a compatible osmoproctectant and a stress-signaling metabolite that accumulates in a variety of plant species in response to environmental stresses (Chaves et al., 2003; Krasensky and Jonak, 2012). Its accumulation in plants is usually coupled with increases in its precursor glutamic acid, ornithine and arginine (Ashraf and Foolad, 2007). As expected and in agreement with previous studies (Verslues and Sharma, 2010), we observed an increase in proline concentration in shoots and roots of non-inoculated plants in response to drought stress (**Figure 9B**). However, inoculation with *B. subtilis* had the reverse effect to decrease proline concentration in shoots and roots of drought-stressed plants at both harvests (**Figures 6** and **7**). While the accumulation of proline in plants is ubiquitous in response to stress, it is still controversial if its presence is an adaptive trait that confers superior stress tolerance or if its accumulation is a symptom of stress damages (Ashraf and Foolad, 2007). A decrease in proline in presence of *B. subtilis* could thus be indicative that there is less damage in drought-stressed timothy in presence of the endophytes.

#### Serine Family

Serine is a precursor of glycine which could be methylated in the organic osmolyte glycine betaine. Glycine betaine accumulation is a widespread response that may protect plants against environmental stress (Chen and Murata, 2011). We observed an increase in serine concentration in response to inoculation with B26 in both drought-stressed and WW plants. However, serine increase did not translate in an increase in glycine concentration that remained stable under all treatments (**Figures 6** and **7**) and thus could not have contributed to the improvement of drought stress tolerance through the synthesis of glycine betaine. The increased serine pool induced by inoculation with B26 could be dedicated to the neo-synthesis of proteins or to the synthesis of purines and pyrimidines (Ros et al., 2014). Studies on drought–stressed Bermuda grass and pearl millet also showed that glycine content in different plant tissues was not affected by drought (Barnett and Naylor, 1966; Kusaka et al., 2005).

#### *γ*-Aminobutyric Acid (GABA)

Rapid accumulation of *γ*-Aminobutyric Acid (GABA) in stressed tissue is thought to be involved in enhanced resistance by providing a critical link in the chain of events leading from perception of environmental stresses to timely physiological responses (Kinnersley and Turano, 2000). We observed an increased accumulation of GABA in stressed shoots and in stressed and non-stressed roots in presence of the endophyte (**Figures 6** and **7**). GABA accumulation could have played a role in the improvement of drought tolerance of timothy inoculated with B26. Levels of *α*-Aminobutyric acid (AABA) an isomer form of the bioactive *β*-aminobutyric acid (BABA) also involved in drought protection were unchanged. Similarly, pre-treatment of *Arabidopsis* with AABA failed to induce drought tolerance (Jakab et al., 2005).

### Plants Response to Drought Stress and Contribution to Osmolytes Pool from *B. subtilis* B26

Plant response to drought stress in absence of endophyte inoculation was evaluated in order to differentiate the plant response from the effect of the endophyte. We observed changes in the concentration of various key metabolites that are typically related to drought tolerance. For instance, total carbohydrates including fructans increased in roots of drought-stressed plants (Van den Ende, 2013) as well as proline in roots and leaves (Chaves et al., 2003) (**Figure 9B**). Among the metabolites that increased in timothy in response to drought stress and are linked to an improvement of plant tolerance are fructan and total carbohydrates in roots and alanine and starch in roots, some were further increased in the presence of the endophyte like asparagine and sucrose in roots (**Figure 6**). Taken together, these observations indicate that B26 likely has the ability to play a role in the metabolic pathways leading to the synthesis of these metabolites-biomarkers and that it is partly through these mechanisms that B26 improves drought stress tolerance in timothy.

It should be taken into account that plant endophytes may also exude osmolytes in response to stress, which may act synergistically with plant-produced osmolytes and stimulate growth under stressed conditions (Madkour et al., 1990; Paul and Nair, 2008). Our study did not discriminate between osmolytes from plant origin or from *B. subtilis*. Thus, the increase in certain osmolytes in drought-stressed inoculated timothy plants could, in part, have originated by the endophyte itself. Under our experimental conditions, how much of the endophyte-derived osmolytes contributed to the total pool of osmolytes is difficult to predict but we could safely mention that the increases are relatively small considering that B26 abundance inside stressed and not stressed timothy tissues remained the same (**Figure 2**).

## Conclusion

According to our knowledge, this is the first study monitoring carbohydrates and amino acids in a cool-season grass colonized by a bacterial endophyte during an extended drought period (up to 8 weeks of withholding water). We observed better agronomical traits such as higher dry weight of shoot and root, and higher photosynthetic rate and stomatal conductance in inoculated plants after 8 weeks of drought stress. Our results clearly show that strain B26 sped up timothy responses to drought stress by increasing the accumulation of either acquired or inducible metabolites associated to drought protection compared to non-inoculated plants. Increases in soluble sugars such as sucrose, fructans and glucose under drought conditions were directly linked to the presence of B26. The majority of amino acids were produced at greater quantities in bacterized timothy under water stress with notable increases in asparagine in shoots, and valine, leucine and isoleucine in shoots and roots, mainly in harvest 1. Therefore this phenomenon may partly explain how *B. subtilis* B26 improves timothy tolerance to drought stress as shown by the observed higher biomass.

## Author Contributions

FG-B conceived, designed and executed the experiments and wrote the first draft of the manuscript. AB and AC provided advice on the experimental design and on the amino acid and sugar analyses. KA performed the multivariate statistical analysis. SJ took part in the experimental design and corrected several drafts of the manuscript. AB, AC, KA corrected the last version of the manuscript.

## Conflict of Interest Statement

The authors declare that the research was conducted in the absence of any commercial or financial relationships that could be construed as a potential conflict of interest.

## Supplementary Material

TABLE S1 **Soluble sugar and amino acid retention times**.

TABLE S2 **Concentrations of metabolites in timothy shoots and roots inoculated (ENDO) or non-inoculated (NE) with B. subtilis B26 after 4 weeks (First harvest) and 8 weeks (second harvest) under well-watered (WW) or water-stressed (DRY) treatments, n = 4**.

FIGURE S1 **Partial least squares-discriminant analyses (PLS-DA) PC1/PC2 score plots for **(A)** Non-inoculated and inoculated plants **(B)** water-stressed (DRY) and WW conditions and **(C)** Harvest 1 and Harvest 2**. The ellipse represents the Hotelling T^2^ with 95% confidence interval. Four biological replications each consisting of ten plants were performed per treatment [Q_(cum)_^2^; cumulative fraction of the total variation of the *X*’s that can be predicted by the extracted components, *R^2^X* and *R^2^Y*; the fraction of the sum of squares of all *X*’s and *Y*’s explained by the current component, respectively].

FIGURE S2 **Representative **UPLC-**ELSD chromatogram of timothy shoots’ amino acids under water-stressed **(A)** and well-watered **(B)** conditions, and of a mixture of 21 amino acids (40 pmol) (C)**. Annotation of metabolites is displayed. See legend of **Figure 6** for abbreviations.

FIGURE S3 **Representative UPLC-ELSD chromatogram of timothy shoots’ carbohydrates under water-stressed **(A)** and well-watered **(B)** conditions, and of a mixture of five carbohydrates (C)**. Annotation of metabolites is displayed. See legend of **Figure 6** for abbreviations.
