# Supplementary material for: Alleviation of Drought Stress and Metabolic Changes in Timothy (Phleum pratense L.) Colonized with Bacillus subtilis B26
Source: Front Plant Sci. 2016 May 3;7:584. doi: 10.3389/fpls.2016.00584 (PMC4854170; doi:10.3389/fpls.2016.00584)
Supplement: TABLE S1 — Soluble sugar and amino acid retention times. [file Table_1.DOCX]

**Supplementary Table 1**. Soluble sugar and amino acid retention times

| **Sugars** |  | **Retention time (min)** |
| --- | --- | --- |
| Fructose  Glucose  Sucrose  Raffinose  Stachyose |  | 1.84  1.97  2.24  3.14  4.64 |
| **Amino Acids** | **Abbreviation** | **Retention time (min)** |
| Aspartic Acid | Asp | 4.08 |
| Glutamic acid | Glu | 4.48 |
| Asparagine | Asn | 6.34 |
| Serine | Ser | 7.48 |
| Glutamine | Gln | 8.14 |
| Histidine | His | 8.63 |
| Glycine | Gly | 10.66 |
| Threonine | Thr | 11.15 |
| Arginine | Arg | 12.45 |
| Alanine | Ala | 13.79 |
| *γ*-aminobutyric acid | GABA | 14.62 |
| *α*-aminobutyric acid | AABA | 16.74 |
| Proline | Pro | 18.32 |
| Methionine | Met | 19.08 |
| Valine | Val | 19.52 |
| Phenylalanine | Phe | 20.41 |
| Leucine | Leu | 21.36 |
| Isoleucine | Ile | 21.67 |
| Lysine | Lys | 22.35 |
