# Supplementary figures and images for: Alleviation of Drought Stress and Metabolic Changes in Timothy (Phleum pratense L.) Colonized with Bacillus subtilis B26
Source: Front Plant Sci. 2016 May 3;7:584. doi: 10.3389/fpls.2016.00584 (PMC4854170; doi:10.3389/fpls.2016.00584)

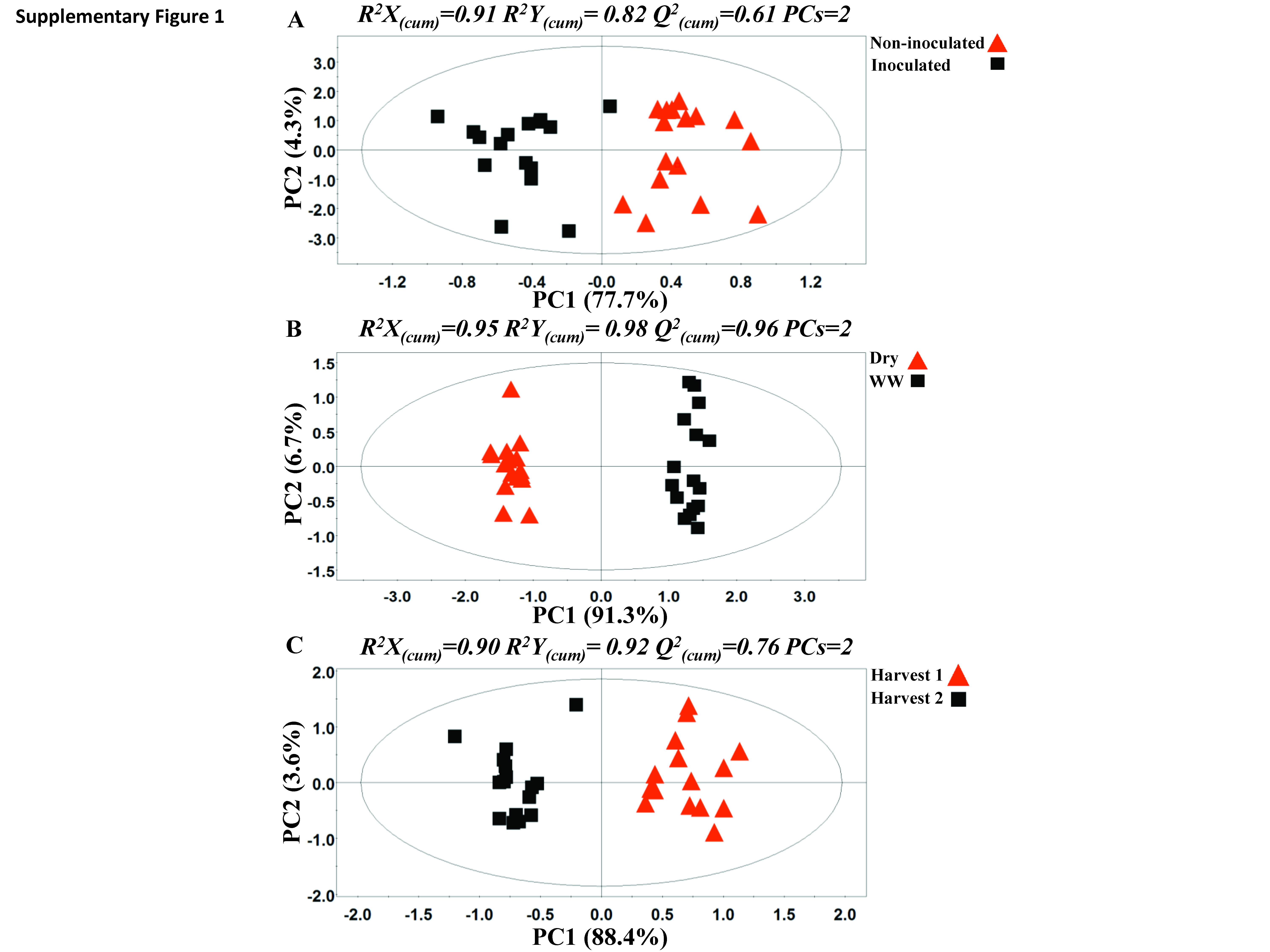

Supplement: FIGURE S1 — Partial least squares-discriminant analyses (PLS-DA) PC1/PC2 score plots for (A) Non-inoculated and inoculated plants (B) water-stressed (DRY) and WW conditions and (C) Harvest 1 and Harvest 2. The ellipse represents the Hotelling T2 with 95% confidence interval. Four biological replications each consisting of ten plants were performed per treatment [Q(cum)2; cumulative fraction of the total variation of the X’s that can be predicted by the extracted components, R2X and R2Y; the fraction of the sum of squares of all X’s and Y’s explained by the current component, respectively]. [file Image_1.TIFF]

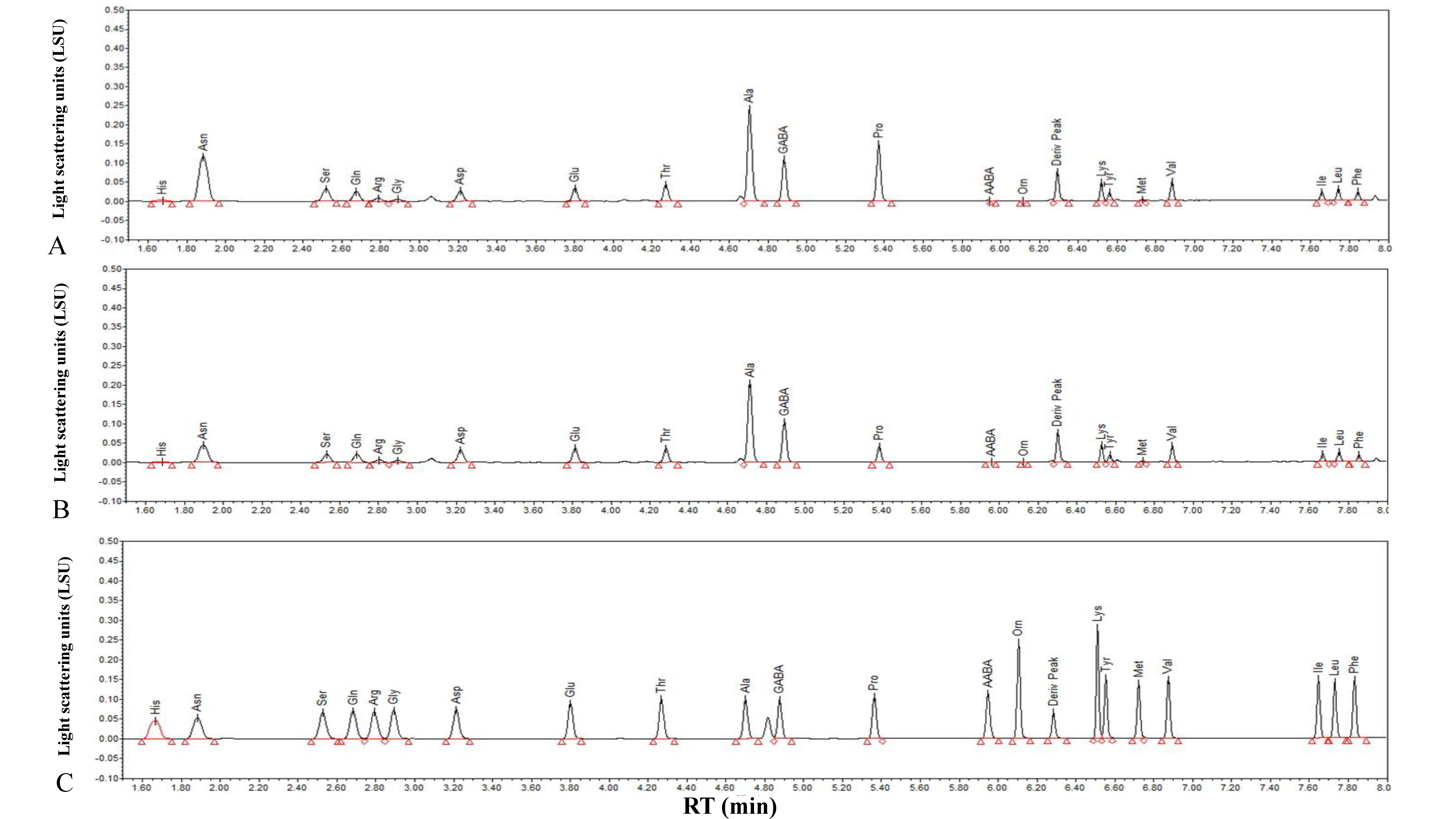

Supplement: FIGURE S2 — Representative UPLC-ELSD chromatogram of timothy shoots’ amino acids under water-stressed (A) and well-watered (B) conditions, and of a mixture of 21 amino acids (40 pmol) (C). Annotation of metabolites is displayed. See legend of Figure 6 for abbreviations. [file Image_2.TIFF]

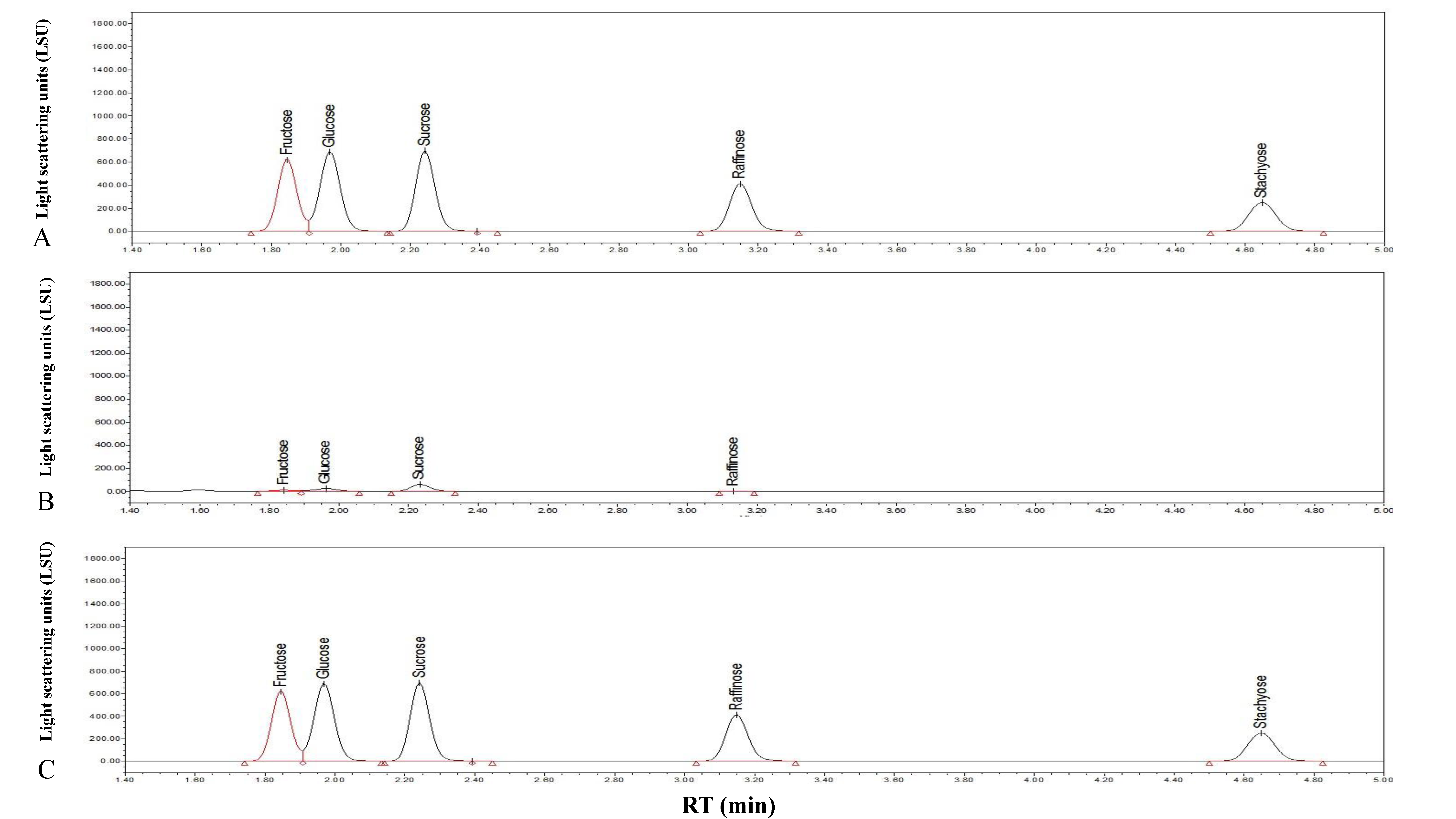

Supplement: FIGURE S3 — Representative UPLC-ELSD chromatogram of timothy shoots’ carbohydrates under water-stressed (A) and well-watered (B) conditions, and of a mixture of five carbohydrates (C). Annotation of metabolites is displayed. See legend of Figure 6 for abbreviations. [file Image_3.TIFF]
